# Rational design of a multi-epitope vaccine targeting the stress-associated proteins for *Mycoplasma fermentans* via immunoinformatics

**DOI:** 10.3389/fbinf.2026.1994081

**Published:** 2026-10-21

**Authors:** Jinjiang He, Jing Mi, Hongmei Tang, Ziyi Zhang, Zhixi Chen

**Affiliations:** 1 Institute of Pathogenic Biology, Basic Medical School, Hengyang Medical College, Key Laboratory of Special Pathogen Prevention and Control of Hunan Province, University of South China, Hengyang, China; 2 Infection Control Department, The First Affiliated Hospital, Hengyang Medical College, University of South China, Hengyang, Hunan, China; 3 Department of Blood Transfusion, The First Affiliated Hospital, Hengyang Medical College, University of South China, Hengyang, Hunan, China

**Keywords:** immunoinformatics, multi-epitope vaccine, *Mycoplasma fermentans*, reverse vaccinology, stress-associated proteins

## Abstract

*Mycoplasma fermentans*: (*M. fermentans*) is a clinically important opportunistic pathogen with intrinsic antibiotic resistance, highlighting the need for targeted intervention strategies. To address this, we applied immunoinformatics and reverse vaccinology to develop a multi-epitope vaccine (MEV) directed against its heat shock proteins (HSPs). The final construct comprised six computationally predicted linear B-cell epitopes, four cytotoxic T lymphocyte (CTL) epitopes, and five helper T lymphocyte (HTL) epitopes, sequentially joined by the specific linkers KK, AAY, and GPGPG. For improved antigen presentation, the vaccine was further equipped with a beta-defensin at the N-terminus and a dendritic cell-targeting signal peptide at the C-terminus. The MEV demonstrated promising antigenicity, as well as favorable structural stability and solubility. *In silico* cloning into pET-28a (+) indicated that efficient heterologous expression is feasible. Immune simulations predicted that a three-dose immunization schedule would trigger a robust and long-lasting secondary immune response. Additionally, the predicted mRNA secondary structure exhibited high thermodynamic stability, supporting the feasibility of the proposed construct for further expression evaluation. Although experimental validation and clinical evaluation are still required, these computational results strongly support the vaccine candidate’s potential.

## 1 Introduction

*Mycoplasmas* constitute the tiniest and most elementary autonomously replicating prokaryotes, comprising a taxonomically varied and globally distributed assemblage of pathogens distinguished by the absence of a peptidoglycan cell wall, a trait that renders them a considerable menace to human and veterinary health (Qin et al., 2019; Hu et al., 2022; Qiu et al., 2024; Xiu et al., 2024). These microorganisms exhibit parasitic capabilities across an exceptionally broad spectrum of hosts, encompassing humans, livestock, wildlife, arthropods, botanical species, and *ex vivo* cell cultures (Deng et al., 2022; Wang et al., 2023; Qiu et al., 2024; Xiu et al., 2024). Within the human host, select *Mycoplasma* species persist as benign commensal flora, whereas others are causally linked to acute infectious syndromes and subsequent post-infectious sequelae (Jiang et al., 2021; Gan et al., 2023; Qiu et al., 2024). Owing to an array of refined strategies for subverting immunological recognition, *Mycoplasma* infections frequently adopt a protracted, chronic trajectory (Deng et al., 2018; Luo et al., 2023; Wang et al., 2023; Zeng et al., 2025).

*M. fermentans* is classified within the class Mollicutes and has been detected and isolated from various tissues, particularly within the genitourinary tract (Benedetti et al., 2014). This organism is associated with a range of human diseases, including chronic inflammatory conditions such as rheumatoid arthritis, as well as respiratory and urogenital tract infections (Rechnitzer et al., 2011; Sim et al., 2023). Furthermore, it has been linked to the development of human immunodeficiency virus and participates in the pathogenic mechanisms underlying a spectrum of neurological diseases (Bustos-López et al., 2024). Notably, *M. fermentans* inhibits p53 activity and collaborates with Ras in oncogenic transformation (Curreli et al., 2021).

Although *M. fermentans* is recognized as a pathogenic *mycoplasma*, its prevalence remains uncertain. It has been detected in the adult urogenital tract, leading to speculation that sexual transmission may be a potential route (Bustos-López et al., 2024). In clinical practice, macrolides are the first-line drugs for treating *M. fermentans* (Pereyre et al., 2002). Nonetheless, there have been instances of resistant strains that exhibit resistance to macrolides. Vaccination plays an essential role in curbing the proliferation of infectious diseases. Consequently, it is vital to adopt effective vaccination strategies to avert *M. fermentans*.

Currently, there is no specific vaccine available for *M. fermentans*. Significant gaps remain in the research surrounding this pathogen. A novel approach to vaccine development, known as reverse vaccinology (RV), emphasizes that vaccine design can commence from sequence information rather than necessitating the cultivation of the pathogen (Rappuoli et al., 2016). Traditionally, most vaccines are based on live-attenuated or killed microorganisms (Masignani et al., 2002). The advantages of inactivated vaccines are twofold: first, they possess a mature and easily scalable production process, facilitating large-scale manufacturing (Wu et al., 2025). Secondly, employing the complete virus as the immunogen can stimulate a more extensive antibody response (Zhu et al., 2024). In contrast, RV utilizes computational analyses and bioinformatics of pathogen genomes to pinpoint possible vaccine targets, known as epitopes, that are most likely to elicit a robust immune response (Khalid and Poh, 2023). RV offers two significant advantages over traditional vaccine development strategies: the identification of candidate antigens does not require the cultivation of the pathogen, and antigens can be identified independently based on purified quantities, making them suitable for vaccine testing (Roachford et al., 2017). RV has been successfully employed to identify candidate antigens for various pathogens, including *Ureaplasma urealyticum*, *Streptococcus anginosus, Neisseria gonorrhoeae*, *Treponema pallidum* and *Streptococcus pneumoniae* (Nahian et al., 2023; Williams et al., 2024; Jiang et al., 2025; Xu et al., 2025; Xu et al., 2026). Despite these advancements, research on the RV of *M. fermentans* remains exceedingly limited.

Within host immune cells, such as macrophages and dendritic cells, *M. fermentans* encounters various stress conditions (Ji et al., 2019). Current research indicates that the bacterial HSPs system is a key factor in its response to external stress, facilitating immune evasion and sustaining intracellular survival (Zügel and Kaufmann, 1999a). As essential components of the HSP70 chaperone system, DnaK and DnaJ play central roles in assisting proper protein folding, preventing aggregation, and responding to thermal and other stimuli (Curreli et al., 2022). Recent research has shown that DnaK and DnaJ originating from different pathogenic bacteria, assist in the adaptation of these microorganisms to the intracellular conditions of the host. Additionally, they serve as significant pathogen-associated molecular patterns (Chan and Groisman, 2024). These proteins engage with pattern recognition receptors located on the surface of dendritic cells, influencing the release of pro-inflammatory cytokines and thus playing a role in the advancement of disease (Wu et al., 2017; Sasaki et al., 2021). Although HSPs are crucial for pathogen survival under host stress conditions, they are highly immunogenic (Verma et al., 2021). Moreover, HSPs can coordinately activate CTLs, HTLs, B-cells, NK cells, and macrophages, leading to a comprehensive immune response (Zhang et al., 2026). Beyond HSPs, bacteria also rely on other stress-associated system to maintain cellular homeostasis under adverse environmental conditions. Among these, the thioredoxin system is an important redox-regulatory system involved in the maintenance of cellular redox balance and the response to oxidative stress. Thioredoxin reductase (TrxR), which catalyzes the NADPH-dependent reduction of thioredoxin, is an essential component of this system (Serrano et al., 2007). In *M. fermentans*, both thioredoxin and thioredoxin reductase have been characterized, and the thioredoxin reductase system has been proposed to contribute to protection against oxidative challenges (Rechnitzer et al., 2011). Therefore, in addition to HSPs, TrxR may also be considered a stress-associated protein relevant to the survival and adaptation of *M. fermentans* under host-associated stress conditions (Ben-Menachem et al., 1997). Additionally, vaccines designed through RV are constructed by selecting components that possess strong antigenicity, high immunogenicity, and non-toxicity (Alnajran et al., 2025). This study primarily focuses on the stress-associated proteins of *M. fermentans*. The current research focuses on developing an MEV derived from the stress-associated proteins of *M. fermentans*, employing computational methods to pinpoint the most immunogenic epitopes. This strategy could lead to the production of an efficient vaccine that offers durable defense against infections caused by *M. fermentans*.

## 2 Methods

The flowchart for the multi-epitope vaccine targeting the stress-associated proteins of *M. fermentans* designed in this study is shown in Figure 1.

**FIGURE 1:**
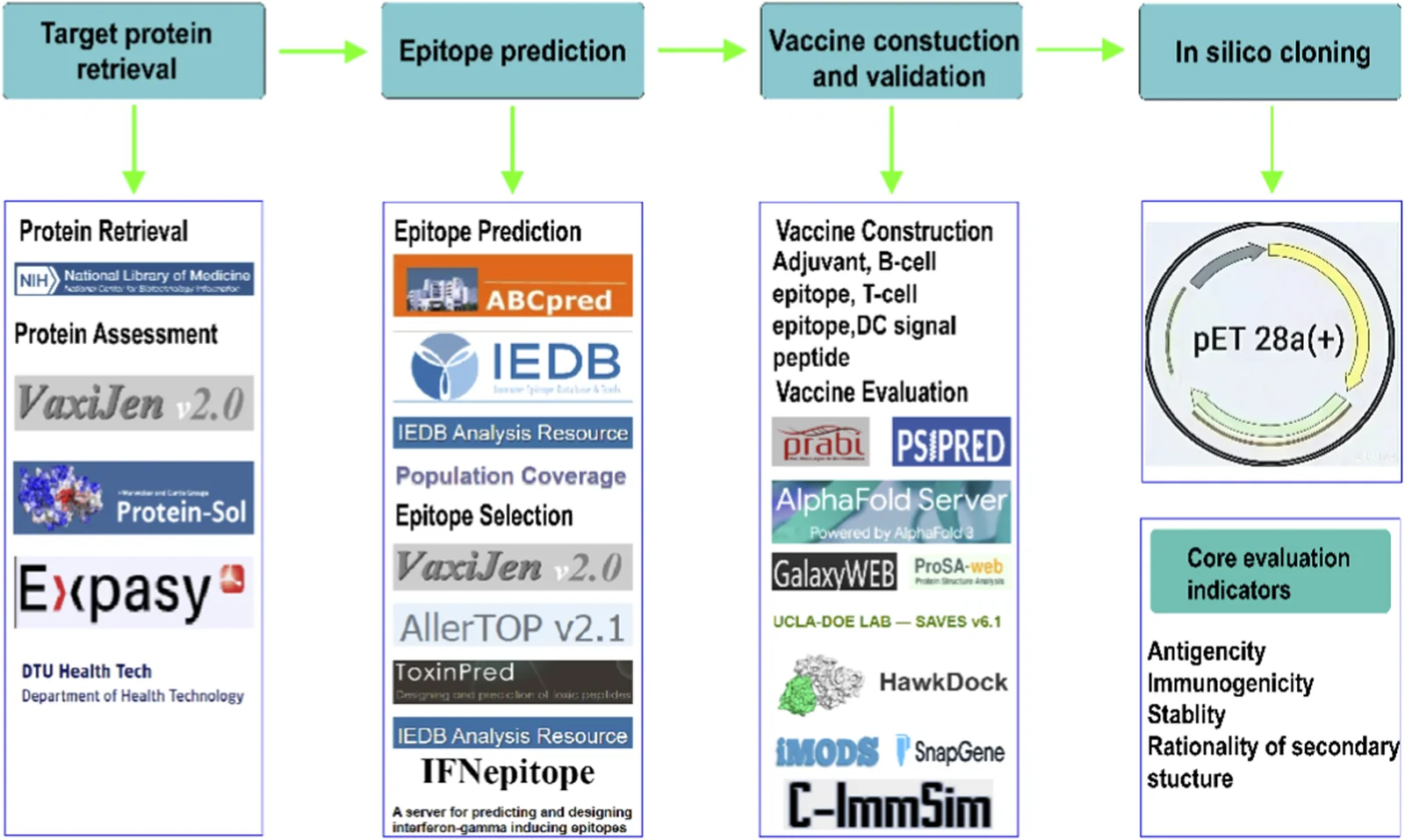
Flowchart of multi-epitope vaccine design.

### 2.1 Acquisition of proteins

The FASTA-formatted amino acid sequences of the stress-associated proteins from *M. fermentans* (M64 strain) were obtained from the NCBI database (GenBank: CP002458.1). Afterward, we assessed various characteristics of these proteins, such as antigenicity, solubility, toxicity, and instability index. The antigenicity of the proteins was assessed via the VaxiJen v2.0 server (Doytchinova and Flower, 2007). For analyzing protein solubility, we utilized the Protein-Sol server (28). The toxicity of the proteins was determined through the VirulentPred server, and the instability index for the proteins was predicted using the Expasy server (Wilkins et al., 1999; Garg and Gupta, 2008). Ultimately, we selected proteins with an antigenicity prediction score >0.4, a solubility prediction score >0.45, no predicted toxicity, and an appropriate molecular weight (Doytchinova and Flower, 2007; Hebditch et al., 2017).

### 2.2 Forecast of epitope

#### 2.2.1 Forecast of B-cell epitopes

To identify B-cell epitopes that exhibit both high efficacy and safety, a prediction was performed. The initial step involved submitting the protein sequences to the ABCpred server, from which epitopes scoring above 0.8 were chosen (Saha and Raghava, 2006). Additional analyses were carried out on these selected epitopes, including antigenicity prediction using VaxiJen 2.0, allergenicity prediction via AllerTOP 2.0, and toxicity prediction with the ToxinPred server (Dimitrov et al., 2014; Rathore et al., 2024). Following these screenings, the optimal epitopes were selected for subsequent vaccine construction.

#### 2.2.2 Forecast of CTL epitopes

To forecast MHC I epitopes of the stress-associated proteins of *M. fermentans*, we utilized the IEDB MHC I binding server (Andreatta and Nielsen, 2016). The NetMHCpan 4.1 EL method, which was the preferred epitope predictor as of September 2023, was employed. Epitopes with a median binding percentile rank that fell below 0.5 were selected for further investigation. At the same time, the immunogenicity of these identified epitopes was evaluated using the MHC I Immunogenicity platform (Calis et al., 2013). Epitopes that had an immunogenicity score greater than 0 were included for the subsequent stage of analysis (Yuan et al., 2024).

#### 2.2.3 Forecast of HTL epitopes

To examine the stress-associated proteins of *M. fermentans* and forecast MHC II epitopes, we utilized the IEDB MHC II binding server (Andreatta and Nielsen, 2016). We applied the NetMHCpan 4.1 EL method, recognized as a reliable predictor for epitopes as of September 2023. Only those epitopes that exhibited a median binding percentile rank below 0.5 were chosen for further examination, with lower percentile ranks indicating stronger predicted binding. Moreover, the assessment of immunogenicity in these epitopes was conducted using the MHC II immunogenicity platform, which stipulates that only epitopes with an immunogenicity score exceeding 0 were considered for additional analysis (Calis et al., 2013; Yuan et al., 2024). The capability of MHC II epitopes to stimulate interferon-gamma (IFN-γ) was investigated using the IFN epitope server (Calis et al., 2013). For the following research, our attention was directed towards epitopes recognized as having the potential to induce IFN.

#### 2.2.4 Coverage of the population

Notable differences in the expression levels of various HLA alleles were observed among diverse ethnic groups around the globe. We examined MHC class I and class II molecules and evaluated their population coverage with the IEDB population coverage tool (Bui et al., 2006).

### 2.3 T-cell epitope-MHC molecular interactions

To predict the immunogenic potential of candidate epitopes, molecular docking was performed to characterize the binding interactions of T-cell epitopes with MHC alleles. Three-dimensional (3D) structural conformations of the target T-cell epitopes were generated in PDB files via the PEP-FOLD3 online platform (Lamiable et al., 2016). Concurrently, the corresponding PDB files of HLA alleles were downloaded from the RCSB Protein Data Bank (RCSB PDB) (Rigden and Fernández, 2023). Water molecules were removed from the HLA structures using PyMOL, and molecular docking analyses of each T-cell epitope with its matched HLA allele were then conducted using the HawkDock online platform, with the docking restricted to the classical peptide-binding groove (Weng et al., 2019).

### 2.4 Vaccine development and verification

#### 2.4.1 Vaccine design

Epitopes were chosen from the previously mentioned pool for vaccine design based on several criteria, including strong antigenic properties, high immunogenic responses, non-toxicity, and a lack of allergenic potential (Solanki et al., 2019). The use of linkers is also of paramount importance. Flexible linkers like GPGPG and AAY enhance solubility and allow for greater conformational adaptability, while the rigid linker EAAAK serves to maintain spatial separation and ensure proper folding (Cernuto et al., 2025). B-cell epitopes were linked with the KK connector; meanwhile, CTL epitopes were connected via the AAY linker, and HTL epitopes were linked via the GPGPG linker (Alexander et al., 1998; Rawal et al., 2021). The beta-defensin (Uniprot accession: P81534) was used as an adjuvant to amplify the immune response (Xu et al., 2026). The adjuvant was subsequently bound to the N-terminus of the vaccine construct via the EAAAK linker. This arrangement minimizes interactions with other biomolecules through effective separation, thereby improving the vaccine’s stability (Arai et al., 2001). Finally, a dendritic cell signaling peptide was linked to the C-terminus of the epitope sequence to improve antigen presentation (Liu et al., 2024).

#### 2.4.2 Vaccine properties

Initially, the sequence of the vaccine was analyzed to forecast its antigenic characteristics, solubility, and possible allergenic potential utilizing the VaxiGen, Protein-Sol, and AllerTOP v2.0 platforms, respectively. Following this, the ProtParam tool was used to assess multiple physicochemical properties of the protein, covering molecular weight, theoretical isoelectric point (pI), and the grand average of hydropathicity (GRAVY), among others. All the above metrics can provide a theoretical basis for subsequent research (Wilkins et al., 1999).

#### 2.4.3 Assessment of secondary and tertiary structures

Protein secondary structure is predominantly defined by the formation of α-helices and β-sheets, which are the most common structural motifs in globular proteins. To characterize the secondary structural properties of the designed multi-epitope vaccine, two widely used online prediction servers, PSIPRED and SOPMA, were employed as previously described (Geourjon and Deléage, 1995; Buchan and Jones, 2019). The protein sequence of the candidate vaccine was rendered to both platforms, and comprehensive quantitative and qualitative analyses of α-helix and β-sheet contents were subsequently obtained. For constructing a 3D model of the vaccine, the sequence was additionally processed through the AlphaFold prediction framework (Varadi et al., 2022). The resulting tertiary structure underwent refinement through the GalaxyRefine web server to improve local stereochemical fidelity. Finally, the overall model quality of the optimized conformation was evaluated with the ProSA-web service (Wiederstein and Sippl, 2007).

#### 2.4.4 Prediction of structural B-cell epitopes

The 3D structure of the vaccine was uploaded to the ElliPro platform to identify structural B-cell epitopes. This server depicts the protein structure in the form of an ellipsoid and determines the protrusion index for the protein residues located outside the ellipsoid (Ponomarenko et al., 2008). Ultimately, continuous epitopes with a score exceeding 0.6 were chosen.

#### 2.4.5 Cloning construction and *in silico* codon refinement

With the aim of enhancing its expression in *E. coli K12*, the MEV underwent codon optimization (Jiang et al., 2025). The Optimizer online tool was utilized accordingly, with optimization parameters set to a GC proportion of 30%–70% and a Codon Adaptation Index (CAI) value between 0.8 and 1.0 (Puigbo et al., 2007). The refined nucleotide sequence was subsequently integrated into the pET-28a (+) expression vector via SnapGene software. Additionally, the restriction enzyme recognition sites for *BamHI* and *XhoI* were introduced at the 5′ and 3′ termini of the target gene fragment, respectively (Fathollahi et al., 2021).

#### 2.4.6 Immune simulations

The C-ImmSim online platform was utilized to assess and model the host immune response elicited by the candidate vaccine within a host organism. This tool gauges the reliability of vaccine design by forecasting the production of antibodies, interferons, and cytokines—the latter being essential for immune regulation—resulting from the interaction of the vaccine with the host (Rapin et al., 2011). A step-based model was established, with each simulation step representing 8 h *in vivo*. To simulate the vaccination schedule, simulations were run for 1 step (8 h), 84 steps, and 170 steps, amounting to a total of 1,050 steps. The HLA restriction parameters used in the immune simulation were kept consistent with those used in the preceding T-cell epitope prediction and population coverage analyses. We set the HLA parameters as HLA-A*30:01, HLA-A*11:01, HLA-DRB1*04:05, HLA-DRB1*15:01, HLA-B*08:01, and HLA-B*07:02. Among these, HLA-B*07:02 was retained as the default parameter because HLA-B*08:01 was the only top-ranked HLA-B allele identified among the screened T-cell epitopes. All remaining parameters were kept at default to guarantee result consistency and reliability (Chen et al., 2024).

#### 2.4.7 Molecular docking

The effectiveness of a vaccine-induced immune response in the host hinges on the interplay between the vaccine protein and the host immune cells (Kang and Compans, 2009). We selected TLR2 to perform molecular docking studies with the developed vaccine protein. The rationale for selecting TLR2 lies in its pivotal role in immune activation triggered by the recognition of *M. fermentans* lipoprotein antigens (Seya and Matsumoto, 2002). The structure of the TLR2 (PDB ID: 2Z81) was sourced from the RCSB PDB. For molecular docking analysis, the HawkDock server was utilized in this study. The server forecasted the structures of protein-protein interactions (PPIs) by integrating ATTRACT docking with re-scoring through HawkRank, while important residues were emphasized using MM/GBSA free energy decomposition (Weng et al., 2019). Finally, the visualization of the vaccine-TLR complex structure was carried out using the PyMOL software.

#### 2.4.8 Simulations of molecular dynamics (MD)

Molecular dynamics (MD) simulations were performed using GROMACS 2025.4 with the Amber99SB-ILDN force field (Shaw et al., 2010; Abraham et al., 2015). The protein–protein complex was solvated in a cubic box using the TIP3P water model, with a minimum distance of 1.0 nm between the protein and the box edge. Sodium and chloride ions were added to neutralize the system charge. The prepared system was subjected to energy minimization using the steepest descent algorithm for 50,000 steps, followed by a 100 ps NVT equilibration with the V-rescale thermostat coupling the system temperature to 300 K. Subsequently, a 100 ps NPT equilibration was conducted using the Parrinello-Rahman barostat. Finally, a 100 ns production simulation was carried out. During the simulation, the cutoff distances for both electrostatic and van der Waals interactions were set to 1.0 nm. Long-range electrostatic interactions were treated using the Particle Mesh Ewald (PME) method. Bond constraints were applied using the LINCS algorithm, neighbor searching was performed using the grid search method, and the integration time step was set to 2 fs. Random seeds were set to −1 to generate different initial velocities, and three independent simulations were performed (Donnelly et al., 2021). RMSD, RMSF, SASA, Rg, hydrogen bonds, and free energy landscape (FEL) analyses were calculated using built-in GROMACS modules. In addition to these conventional simulations, the intrinsic flexibility of the vaccine construct was further evaluated using Normal Mode Analysis (NMA) conducted through the iMODS server. This is a server for protein dynamics simulation and conformational analysis. The efficiency and applicability of NMA were enhanced and extended, respectively, through the use of its unique internal coordinate representation (López-Blanco et al., 2014).

#### 2.4.9 Prediction of vaccine mRNA

Following optimization by the Optimizer, the sequence data were provided to the RNAfold web server to forecast the secondary structural conformation of the candidate vaccine mRNA (Gruber et al., 2008).

#### 2.4.10 Human proteome homology analysis and prediction

The complete amino acid sequence of the final MEV construct, provided in FASTA format, was subjected to BLASTp analysis against the *Homo sapiens* Reference proteins database (taxid:9606) using the NCBI BLAST web interface. Significant sequence similarities were evaluated based on E-value, sequence identity, and query coverage (Altschul et al., 1997; Johnson et al., 2008).

## 3 Results

### 3.1 Characterization of HSPs

The physicochemical profiles of the selected stress-associated proteins are detailed in Table 1. As illustrated in Table 1, these proteins display broad antigenic potential, pronounced solubility, a lack of toxicity, an absence of transmembrane helical domains, and no detectable signal peptide cleavage sites. Such characteristics render them particularly amenable to incorporation within vaccine constructs. Furthermore, Figure 2 depicts the modeled 3D conformations of these stress-associated proteins, with the final B- and T-cell epitope positions explicitly indicated.

**TABLE 1:** Summary of physicochemical and antigenic properties predicted for the HSPs.

Protein	Accesion number	Antigenicity	Solubility	Molecular weight (kDa)	Theoretical pI	Instability index	Signal peptide	Topology
DnaK	ADV34134.1	0.52	0.722	64.20	5.07	32.50	0	1
DnaJ	ADV34142.1	0.69	0.635	41.81	9.11	18.59	0	1
Trigger factor	ADV34158.1	0.65	0.579	58.59	8.87	34.33	0	1
Putative heat shock ATP-dependent protease	ADV34408.1	0.51	0.576	113.63	6.60	34.15	0	1
ATPase subunit	ADV35023.1	0.43	0.620	81.22	7.10	39.51	0	1
Thioredoxin reductase	ADV34906.1	0.43	0.592	34.29	8.47	18.01	0	1

**FIGURE 2:**
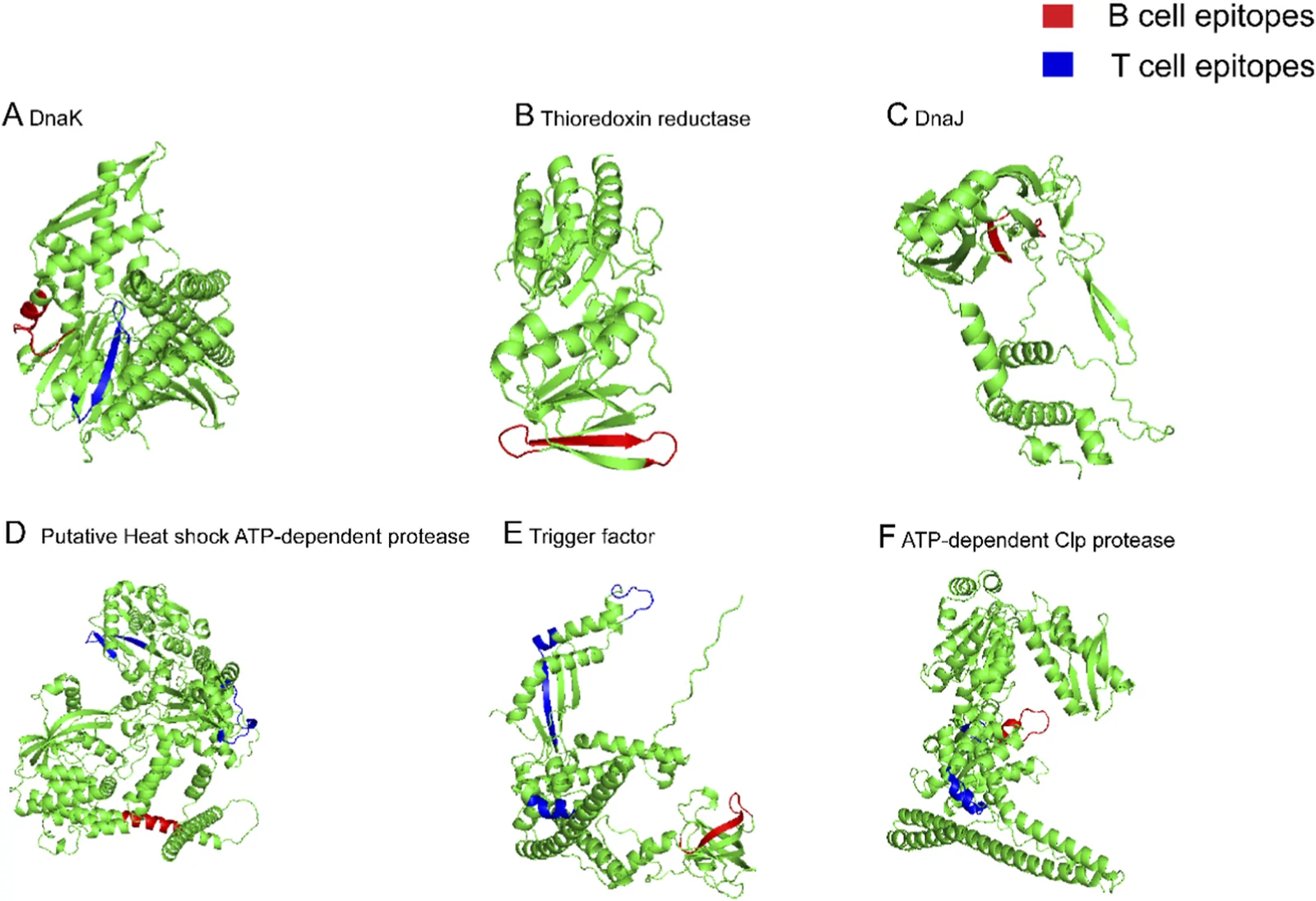
3D configuration of the HSPs in *M. fermentans*. **(A)** 3D structure of DnaK; **(B)** 3D structure of thioredoxin reductase. **(C)** 3D structure of DnaJ. **(D)** 3D structure of putative heat shock ATP-dependent protease. **(E)** 3D structure of trigger factor. **(F)** 3D structure of ATP-dependent Clp protease. The blue structures correspond to T cell epitopes, and the red ones to B-cell epitopes.

### 3.2 Identification and refinement of candidate epitopes

#### 3.2.1 Selection of B-cell epitopes via computational prediction

B-cell epitopes of the target proteins were predicted with the computational tool ABCpred. The candidate sequences obtained were then assessed based on their antigenic properties and allergenic risks. The criteria for selection emphasized epitopes with high ABCpred scores, strong antigenicity, and profiles that are non-toxic and non-allergenic. Based on these stringent filters, a final panel of six B-cell epitopes was retained. A comprehensive summary of their corresponding metrics is provided in Table 2.

**TABLE 2:** List of B-cell epitopes identified from the HSPs.

Protein	Peptide	Position	ABCpred score	Antigenicity
ADV34134.1	LVEKILNRKPNKSVNP	325–340	0.89	0.7438
ADV34142.1	GDKIKIDGYGEKGRNG	235–250	0.88	2.0779
ADV34158.1	PFDGGEAQGFDLKLGS	224–239	0.89	1.8890
ADV34408.1	KEVAKEYNEENEKTKK	381–396	0.91	1.3040
ADV35023.1	HMLIGTGRNAEGGMDA	201–216	0.87	1.1115
ADV34906.1	GENQLTSVTIRNKKTN	256–271	0.85	1.1184

#### 3.2.2 Identification of T cell epitopes

We employed the IEDB binding servers for MHC I and MHC II to investigate the CTL and HTL epitopes obtained from the stress-associated proteins of *M. fermentans*. Then, we analyzed the antigenicity, immunogenicity, allergenicity, and toxicity of epitopes that exhibited a median binding percentile rank of less than 0.5. In particular, for HTL epitopes, we also predicted the IFN-γ scores to identify those with a strong potential for stimulating the production of interferon-gamma. In the end, we identified five HTL epitopes and four CTL epitopes. The comprehensive data can be found in Tables 3, 4, respectively.

**TABLE 3:** List of predicted CTL epitopes derived from the HSPs.

Protein	Allele	Position	Peptide	Antigenicity	Immunogenicity	Allergenicity	Toxicity
ADV34158.1	HLA-A*30:01, HLA-A*11:01, HLA-A*03:01	44–52	KIPGFRPGK	1.0236	0.20366	NON-ALLERGEN	Non-Toxin
	HLA-B*08:01, HLA-A*24:02	390–398	KFAFRILSL	1.8478	0.20016	NON-ALLERGEN	Non-Toxin
	HLA-A*68:01	22–30	EINGKEWEK	1.7693	0.16169	NON-ALLERGEN	Non-Toxin
ADV35023.1	HLA-A*11:01, HLA-A*03:01	66–74	LVGEPGVGK	1.1598	0.18749	NON-ALLERGEN	Non-Toxin

**TABLE 4:** List of predicted HTL epitopes derived from the HSPs.

Protein	Allele	Position	Peptide	Antigenicity	IFN-g scores	Adjusted rank
ADV34134.1	HLA-DQA1*01:02/DQB1*06:02	11–25	LGTTNSAVAIVDGGT	0.4821	1	0.08
ADV34158.1	HLA-DRB1*04:05 HLA-DRB1*04:01	16–30	ELVYTLEINGKEWEK	1.1386	0.266	0.44
ADV34408.1	HLA-DPA1*02:01/DPB1*01:01, HLA-DPA1*03:01/DPB1*04:02, HLA-DPA1*01:03/DPB1*04:01	711–725	QAGLKKEQFNIDDKT	0.9699	1	0.17
	HLA-DQA1*04:01/DQB1*04:02	811–825	IEVSSFKSKNSGIKL	1.3055	1	0.23
ADV35023.1	HLA-DQA1*01:01/DQB1*05:01, HLA-DRB1*15:01 DQA1*01:02/DQB1*06:02	216–230	IKDRFENYHNVKIQD	0.8377	1	0.28

#### 3.2.3 Coverage of the population by T cell epitopes

The estimation of T-cell epitope coverage relied on the previously selected four CTL epitopes and five HTL epitopes. Utilizing the IEDB population coverage tool, we conducted predictions across 16 distinct regions. Generally, the coverage rates were predominantly over 98%, whereas the lowest coverage was observed in South Africa, which recorded only 49.73%. Comprehensive data can be found in Figure 3.

**FIGURE 3:**
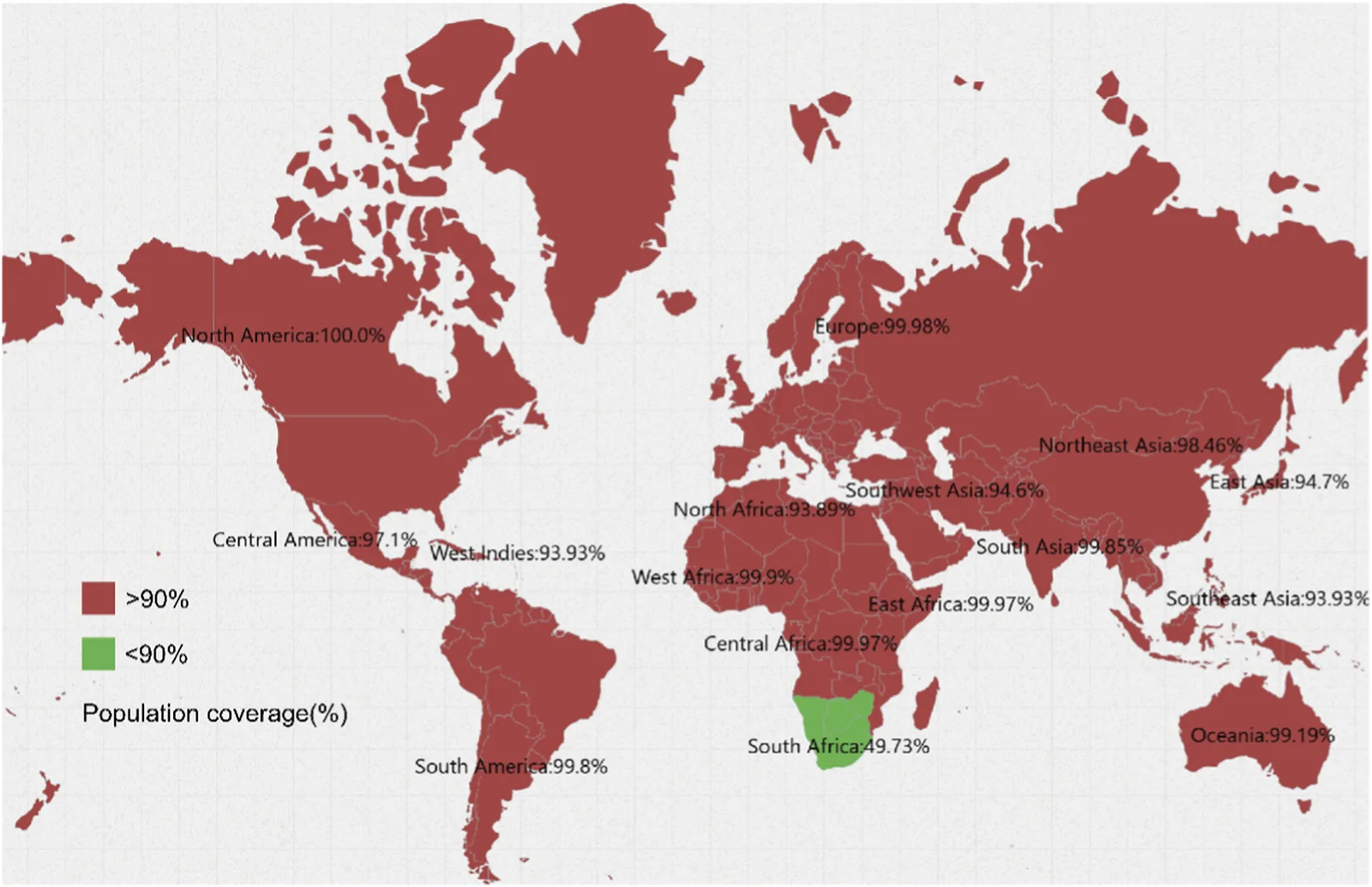
The population coverage of MHC I and MHC II epitopes worldwide.

### 3.3 Molecular interactions between T Cell epitopes and MHC

To predict the interactions between T-cell epitopes and their respective MHC alleles, docking studies were conducted. The coordinates of the HLA molecules utilized in this analysis—specifically HLA-A68:01 (6PBH), HLA-A24:02 (7MJA), HLA-A03:01 (9CX1), and HLA-A11:01 (5WJN)—were obtained from the RCSB PDB. These results are illustrated in Figure 4.

**FIGURE 4:**
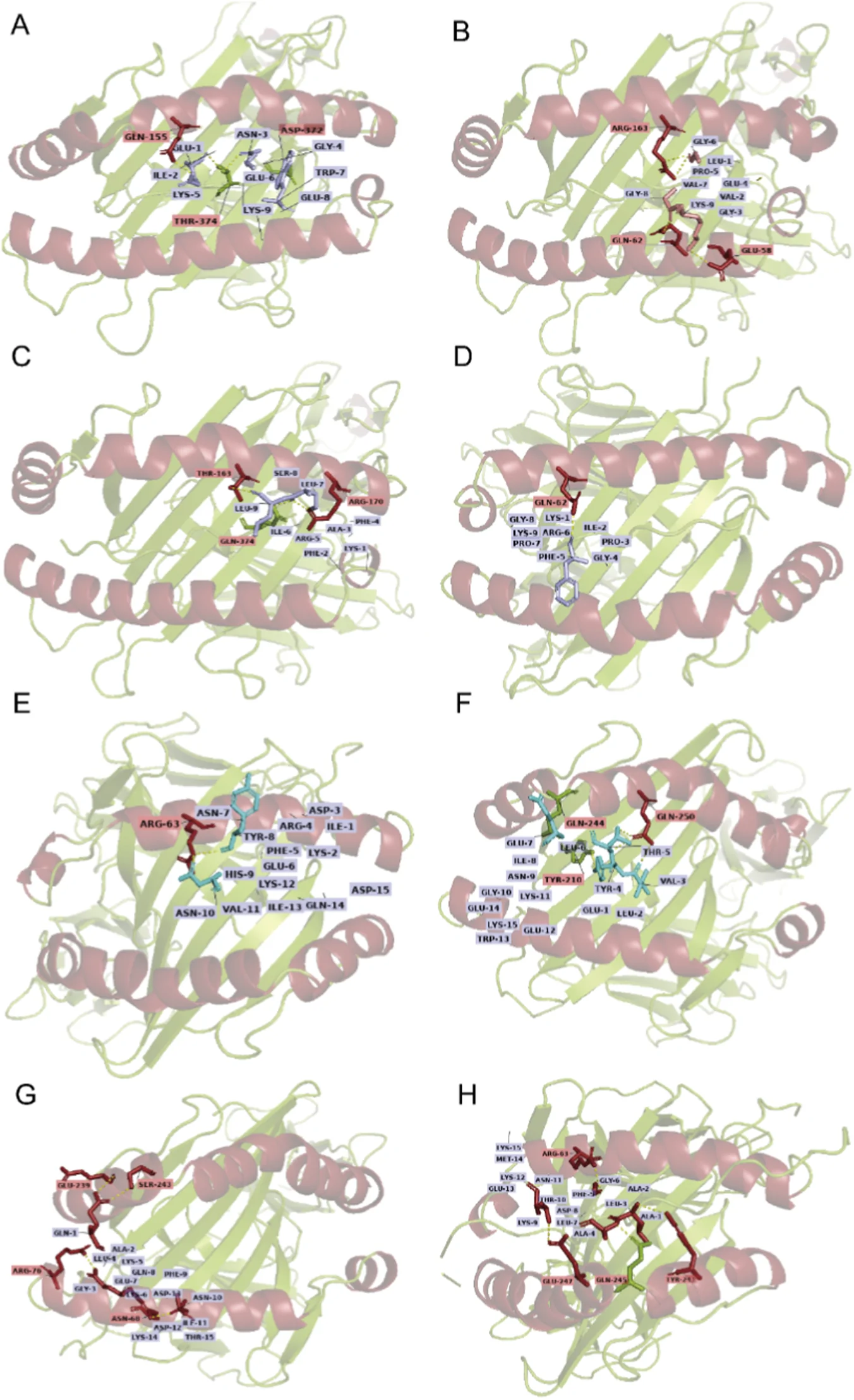
Molecular docking simulations were performed to characterize the interaction modes between T cell epitopes and their corresponding prevalent alleles, with results detailed as follows: **(A)** The CTL epitope EINGKEWEK bound to HLA-A*68:01 yielded a complex with a calculated binding free energy of −35.84 kcal/mol. **(B)** The CTL epitope LVGEPGVGK paired with HLA-A*11:01 resulted in a binding free energy of −17.41 kcal/mol. **(C)** The CTL epitope KFAFRILSL docked against HLA-A*24:02 produced a complex exhibiting a binding free energy of −44.58 kcal/mol. **(D)** The CTL epitope KIPGFRPGK complexed with HLA-A*03:01 displayed a binding free energy of −39.16 kcal/mol. **(E)** The HTL epitope IKDRFENYHNVKIQD in association with DQA1*01:02/DQB1*06:02 gave a binding free energy value of −33.8 kcal/mol. **(F)** The HTL epitope ELVYTLEINGKEWEK interacting with HLA-DRB1*04:01 registered a binding free energy of −27.46 kcal/mol. **(G)** The HTL epitope QAGLKKEQFNIDDKT binding to HLA-DP1 showed a binding free energy of −34.03 kcal/mol. **(H)** The HTL epitope LGTTNSAVAIVDGGT docked with DQA1*01:02/DQB1*06:02 exhibited a binding free energy of −20.97 kcal/mol.

### 3.4 Development of an epitope-based vaccine

#### 3.4.1 Construction of MEV

The selected vaccine construct consists of 318 amino acid residues in total. At the N-terminus, a beta-defensin adjuvant was integrated using a rigid EAAAK linker. Additionally, the design incorporates six B-cell epitopes attached by KK linkers, alongside four CTL epitopes connected with AAY linkers, and five HTL epitopes attached through GPGPG spacers. The immunogenicity prediction by VaxiGen indicated that the MEV sequence was classified as a probable immunogen, with a predicted probability of 100%, and the profile was predicted to be non-allergenic. The Protein-Sol server further estimated a solubility score of 0.729 for this construct. Additionally, using the ProtParam online tool, the calculated molecular weight of the vaccine was 34.6 kDa, which is within the optimal range for protein stability and expression. The instability index was determined to be 17.99, a value that indicates the vaccine possesses favorable structural stability and high resistance to proteolytic degradation *in vivo*. The aliphatic index was 61.07, suggesting thermostability. The GRAVY was −0.8971, denoting a hydrophilic character.

#### 3.4.2 Evaluation of secondary structural components

The 2D structural analysis of the vaccine construct was conducted utilizing the SOPMA and PSIPRED v3.3 web tools. As illustrated in Figure 5, the allocation of secondary motifs consists of 33.33% α-helices, 17.30% extended β-strands, 7.23% beta turn, and 42.14% random coils.

**FIGURE 5:**
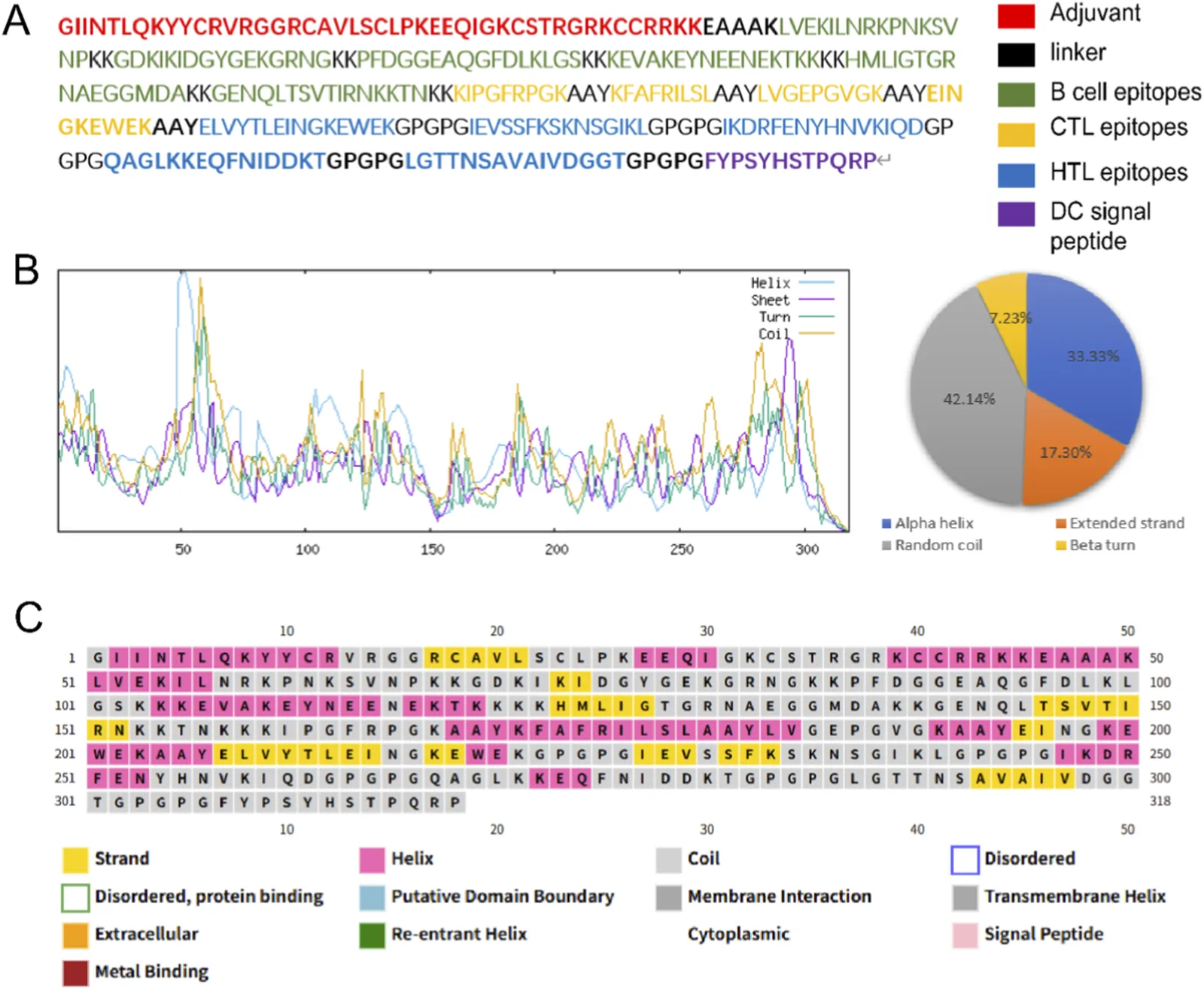
**(A)** Distribution and frequency of amino acid residues comprising the vaccine sequence. **(B)** *In silico* analysis of secondary structural elements as determined by the SOPMA server. **(C)** Validation of secondary structure prediction utilizing the PSIPRED v3.3 algorithm.

#### 3.4.3 Tertiary model construction and refinement

The initial prediction of the 3D structure was accomplished by entering the amino acid sequence into the AlphaFold server. We additionally obtained the pLDDT plot and PAE heatmap for this model. Following this, the preliminary model underwent structural refinement through the GalaxyWEB platform. Figure 6A shows the raw structure predicted by AlphaFold, while Figure 6B presents the 3D model refined by GalaxyWEB. Subsequently, the quality of both structures was validated using three independent computational tools, namely, ProCHECK, ProSA-web, and ERRAT. Evaluation of the Ramachandran plot using ProCHECK indicated a significant improvement in stereochemical quality after refinement with GalaxyWEB. Ramachandran plot analysis of the refined structural model revealed that 95.2% of amino acid residues were located in the most favored core regions, while 4.8% were found in additional allowed areas, with no residues present in generously allowed or disallowed conformations. In comparison, the unrefined AlphaFold model displayed a distribution of 81.0% core, 15.1% allowed, 3.2% generously allowed, and 0.8% disallowed residues.

**FIGURE 6:**
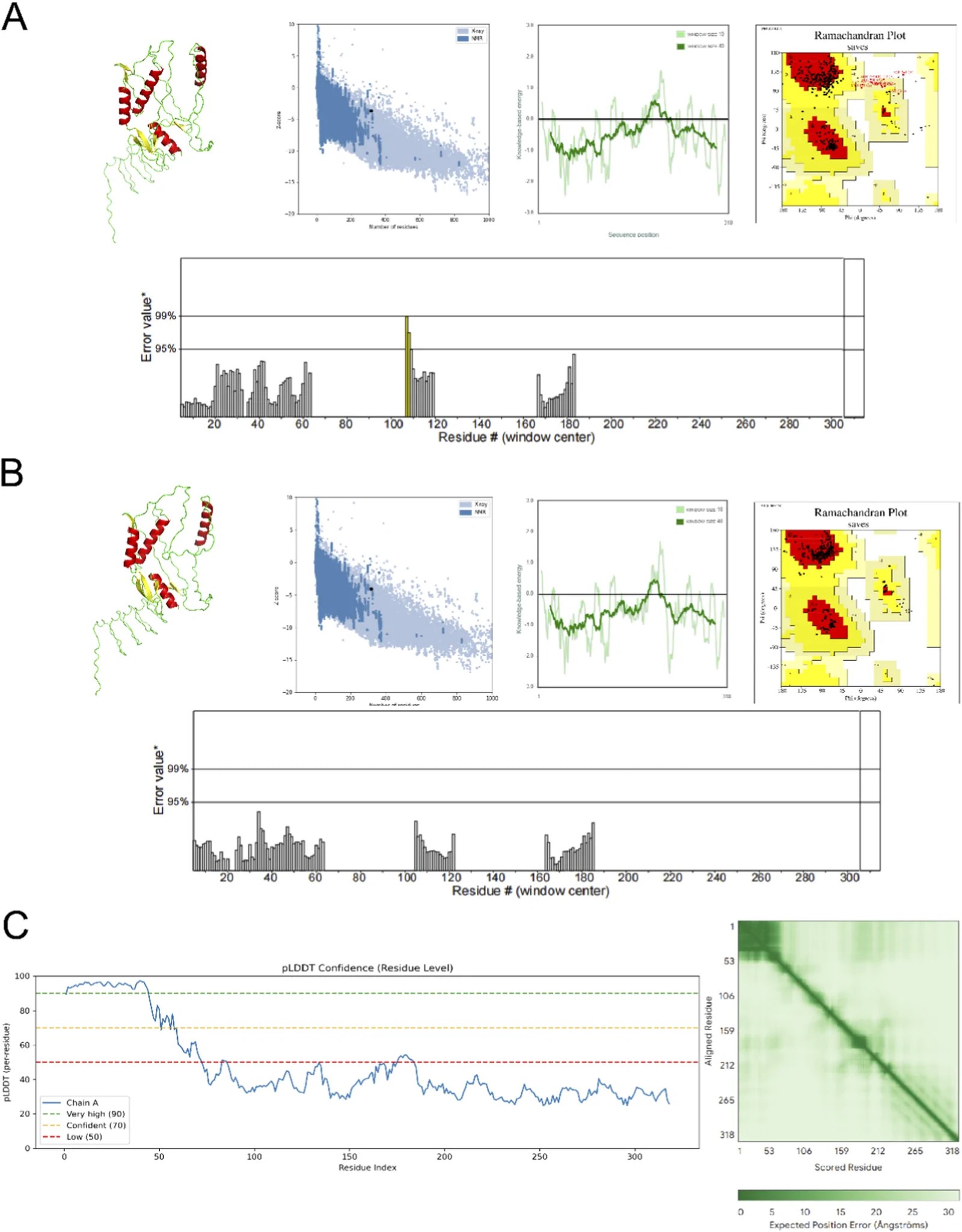
Generation and quality assessment of the tertiary structure models. **(A)** 3D conformation derived from AlphaFold, accompanied by the corresponding Ramachandran plot (PROCHECK), ProSA energy profile, and ERRAT overall quality metric. **(B)** 3D conformation obtained via GalaxyWEB refinement, depicted alongside the associated Ramachandran plot (PROCHECK), ProSA energy diagram, and ERRAT quality evaluation. **(C)** Per-residue confidence plot and PAE heatmap of the AlphaFold model.

Complementary validation metrics further corroborated this improvement. The ProSA-web Z-score shifted from −3.66 to −4.08, indicating enhanced overall model quality relative to native protein conformations. Concurrently, the ERRAT quality factor increased markedly from 97.64 to 100, suggesting the successful optimization of local atomic interactions within the refined structure.

Besides, because the predicted MEV structure was used as the starting model for the subsequent docking and MD analyses, we additionally evaluated its confidence using pLDDT and PAE (Figure 6C). The pLDDT profile was heterogeneous: values were high in the N-terminal region (approximately residues 1–40), declined thereafter, and were generally low across much of the remaining construct. The PAE map also showed higher predicted errors for many nonadjacent residue pairs, indicating uncertainty in the relative positioning of different regions.

#### 3.4.4 Forecast of structural B-cell epitopes

Analysis performed with the ElliPro online platform revealed the presence of four linear structural B-cell epitopes in the vaccine design, with scores of 0.783, 0.761, 0.674 and 0.672, respectively (as shown in Figure 7).

**FIGURE 7:**
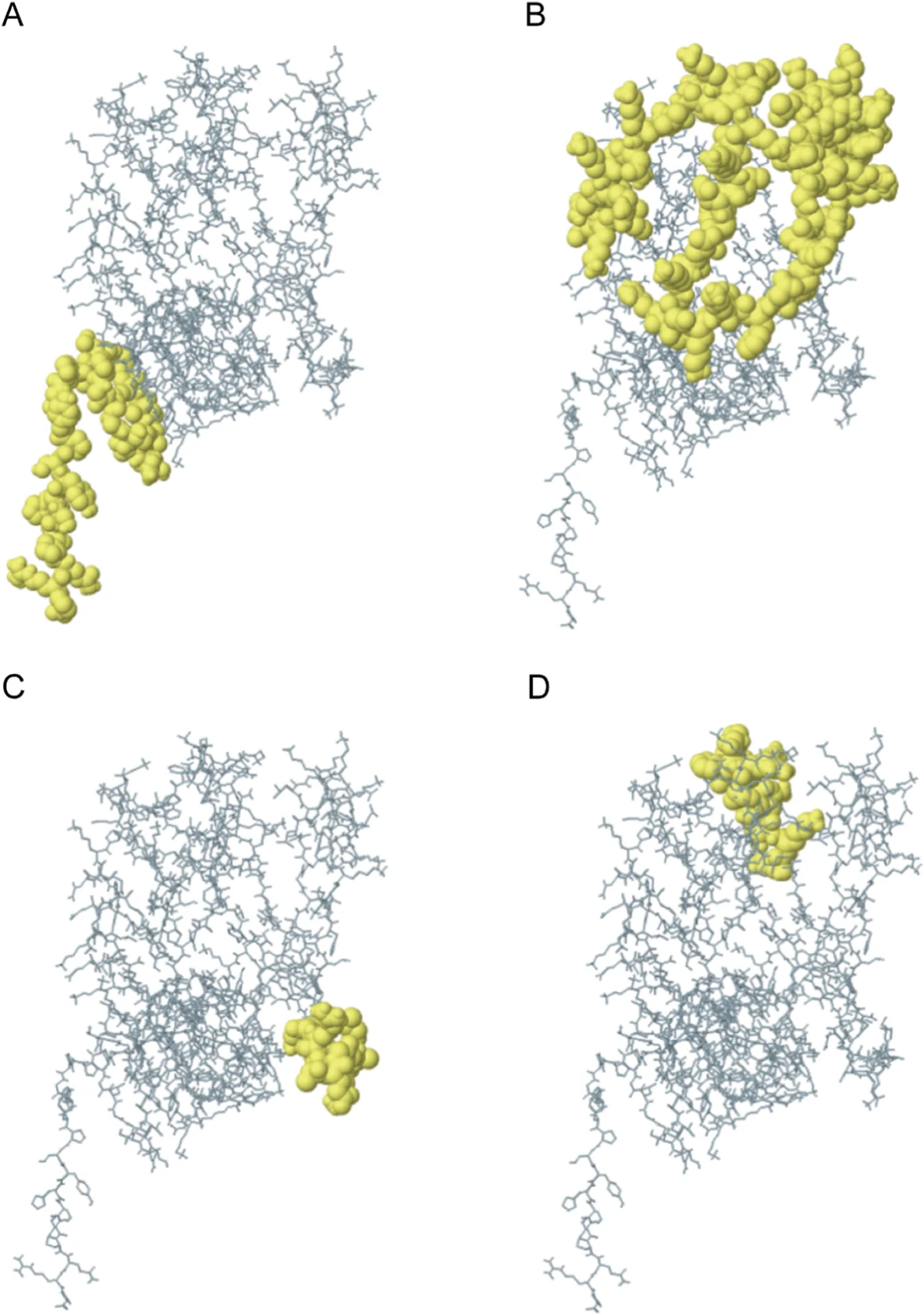
The conformational B-cell epitopes were predicted by ElliPro server. **(A)** AA 285–318: PGLGTTNSAVAIVDGGTGPGPGFYPSYHSTPQRP ElliPro score: 0.783. **(B)** AA 53–121: EKILNRKPNKSVNPKKGDKIKIDGYGEKGRNGKKPFDGGEAQGFDLKLGSKKKEVAKEYNEENEKTKKK ElliPro score: 0.761. **(C)** AA 129–143: GRNAEGGMDAKKGEN ElliPro score:0.674. **(D)** AA 153–165: KKTNKKKIPGFRP ElliPro score:0.672.

#### 3.4.5 Vaccine *in silico* cloning

Following codon optimization for *E. coli* K12 using the Optimizer server, the vaccine sequence (959 nt) possessed a GC content of 49.2% and an optimal CAI of 1.0. The gene termini were then engineered to incorporate *XhoI* and *BamHI* restriction sites. Subsequently, the gene was cloned into the pET-28a (+) plasmid via these sites using SnapGene (Figure 8), yielding a final construct of 6288 bp.

**FIGURE 8:**
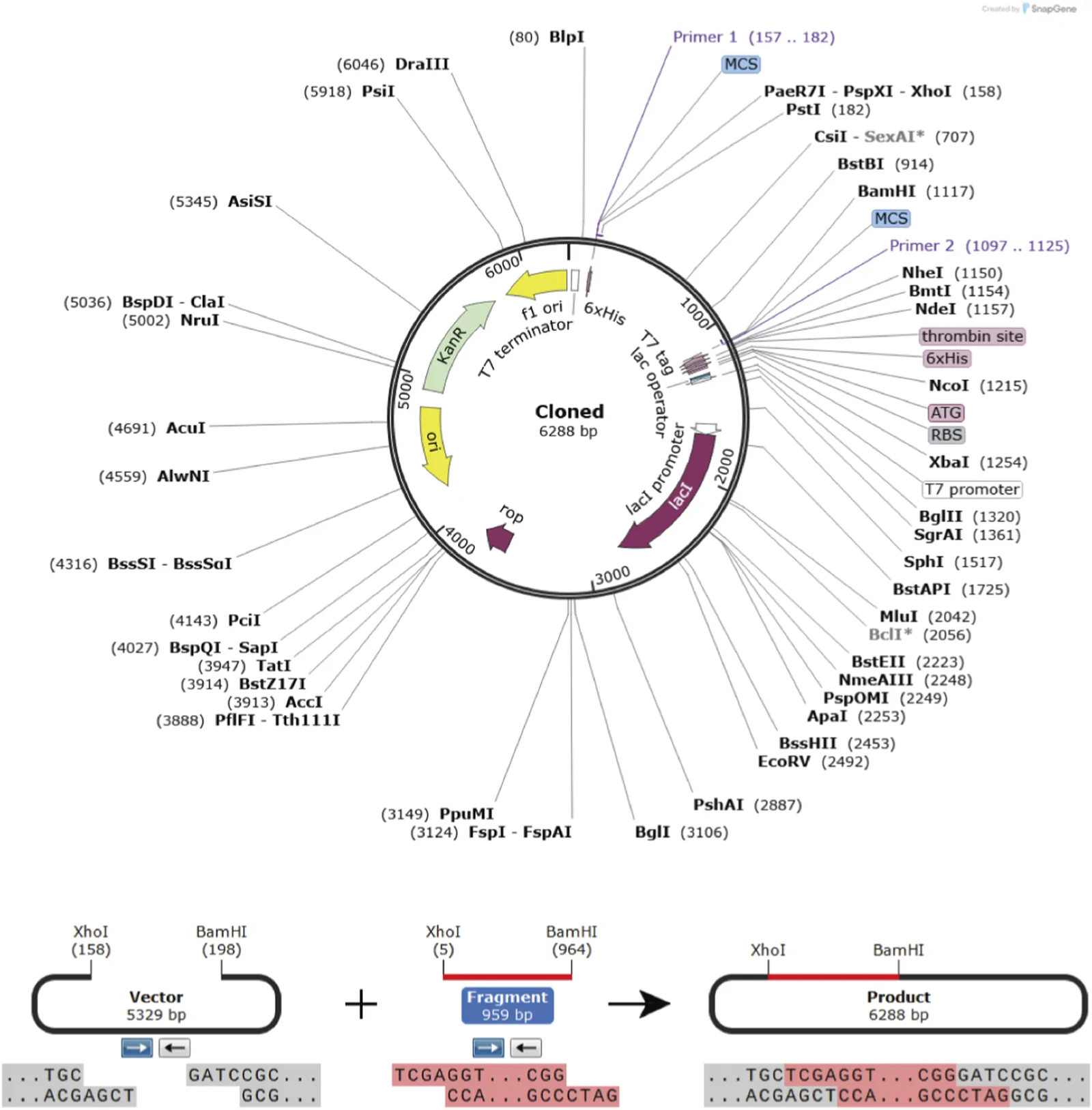
Schematic depiction of the *in silico* insertion of the engineered vaccine construct into the pET28a (+) plasmid. The vaccine coding sequence is highlighted in red, whereas the backbone of the pET28a (+) vector is represented by the black circular element.

#### 3.4.6 Immune simulations

Following vaccination, the designed vaccine construct elicited a predicted activation of multiple immune components. As shown in Figure 9A, the levels of immunoglobulins, including IgM, IgG1, and IgG2, increased markedly during the secondary and tertiary immune responses, accompanied by a gradual decline in antigen levels, suggesting enhanced antigen-specific immune responses and antigen clearance. Figure 9B presents the predicted cytokine response profiles induced by the vaccine construct, indicating the activation of multiple cytokine-mediated immune pathways. The B-cell-mediated immune responses shown in Figure 9C demonstrated an increase in B-cell populations that was maintained at relatively elevated levels. Similarly, T-cell-mediated responses (Figures 9D–F) exhibited sustained activation during the simulated immune responses. In addition, the predicted responses of natural killer cells and macrophages (Figures 9G,H) showed increased immune activity following vaccination. The responses of dendritic cells and epithelial cells (Figures 9I,J) further indicated the potential involvement of antigen-processing and innate immune components. Collectively, these immunological simulation results suggest that the designed vaccine construct has the potential to induce coordinated humoral and cellular immune responses. In summary, our findings suggest that the coordinated action of these cells contributes to the enhancement of overall immune system function.

**FIGURE 9:**
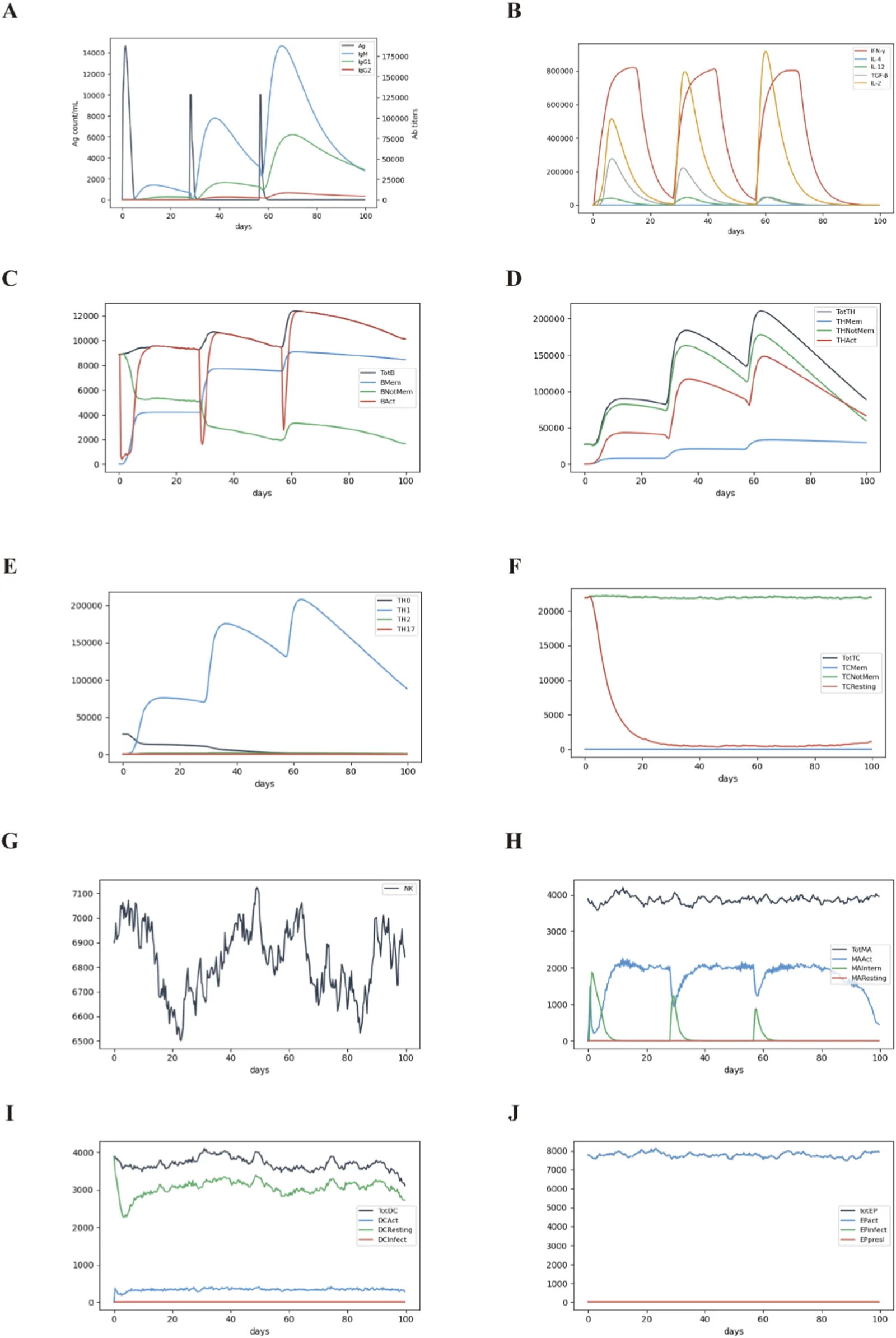
The results of immunological simulations for the designed vaccine construct. **(A)** The responses of immunoglobulins to antigens. **(B)** The levels of immune responses of cytokines. **(C)** B-cell-mediated immune response. **(D–F)** T-cell-mediated immune response. **(G)** The levels of immune responses of natural killer cells. **(H)** The levels of immune responses of macrophages. **(I)** The levels of immune responses of dendritic cells. **(J)** The levels of immune responses of epithelial cells.

#### 3.4.7 Molecular docking

TLR2 was selected as the target immune receptor due to its essential role in mediating the host defense against *M. fermentans.* The interaction between the MEV construct and TLR2 (PDB ID: 2Z81) was evaluated through molecular docking simulations. The HawkDock server was employed to execute the docking calculations and to determine the corresponding binding free energies. A HawkDock score of −5462.62 was recorded for the vaccine–receptor complex. Figure 10 illustrates the 3D conformation of the docking. Computational docking studies afforded a total binding free energy (ΔGtotal) of-44.28 kcal/mol for the intermolecular interaction between the vaccine and TLR2. This value was decomposed into the following energetic contributions: polar solvation energy (ΔGgb), non-polar solvation energy (ΔGsa), electrostatic energy (ΔEelec), and van der Waals energy (ΔEvdw). The detailed values of each individual energy component are provided in Table 5.

**FIGURE 10:**
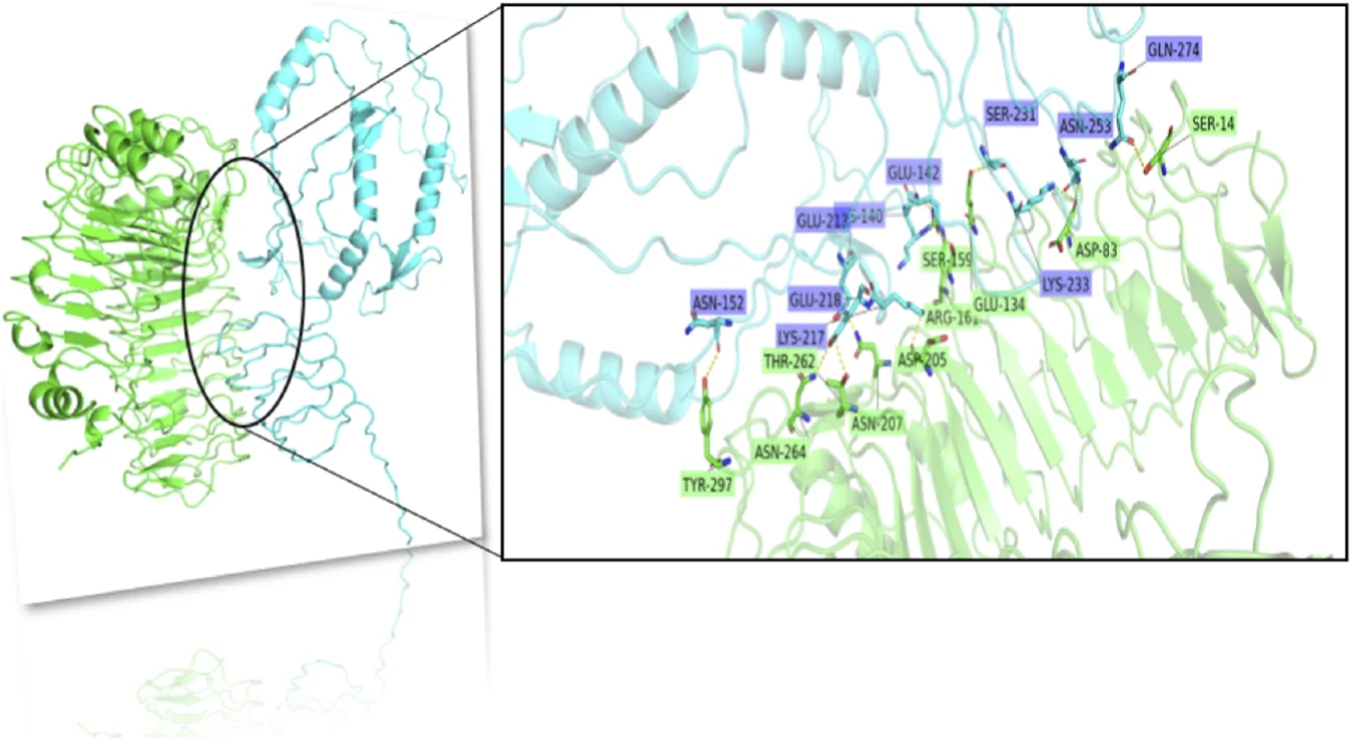
Structural modeling of the vaccine–TLR2 interaction derived from molecular docking. A 3D representation of the vaccine–TLR2 complex obtained via the HawkDock server.

**TABLE 5:** Breakdown of the total MM-GBSA binding free energy for vaccine–TLR2 complex, detailing contributions from individual energy terms.

Energy component	MMGBSA (kcal/mol)
ΔGEvdW	−101.69
ΔGEelec	−1034.98
ΔGGB	1106.61
ΔGSA	−14.22
ΔGTOTAL	−44.28

#### 3.4.8 MD simulations

RMSD was used to assess structural deviations during the simulations. As shown in Figure 11A, all three independent 100-ns trajectories exhibited a rapid RMSD increase during the initial stage, followed by fluctuations within a relatively bounded range. However, the RMSD levels differed among replicates: MD2 remained higher (approximately 1.6–1.7 nm) than MD1 (approximately 1.3–1.4 nm) and MD3 (approximately 1.2–1.3 nm), while MD1 showed a modest gradual increase over the course of the simulation. Thus, the three trajectories showed a similar initial adjustment but did not reach identical RMSD values. Rg was monitored to evaluate the global compactness of the complex. As shown in Figure 11B, Rg decreased from approximately 3.7 nm during the early stage in all three simulations and then fluctuated around approximately 3.3–3.5 nm. The similar overall decrease and subsequent bounded fluctuations across MD1–MD3 suggest a reproducible change in compactness, although the final Rg values varied modestly among replicates. The RMSF profiles showed broadly similar patterns across the three trajectories (Figure 11C). Most atom-index regions exhibited relatively low fluctuations, while higher peaks occurred in corresponding regions, particularly around atom indices 9,000–10,000 and toward the higher-index end of the profile. These results indicate that the overall distribution of flexible regions was similar across replicates, although the magnitude of individual peaks varied. SASA decreased during the early part of all three simulations (Figure 11D), indicating a similar initial reduction in solvent-exposed surface area. The trajectories then fluctuated, with replicate-specific differences becoming more apparent later: MD3 reached lower values toward the end, whereas MD2 increased, and MD1 remained between these profiles. Therefore, the initial SASA trend was reproduced across the three runs, while their later solvent-accessible surface areas were not identical. Hydrogen-bond counts increased overall during the early stage of the simulations and subsequently fluctuated in all three trajectories (Figure 11E). Counts generally ranged from approximately 5–38, reflecting a dynamic hydrogen-bonding network. The late-stage counts differed among replicates, with MD2 showing a decrease toward the end, whereas MD3 maintained comparatively higher counts. These results suggest that hydrogen-bonding interactions persisted during the simulations but varied dynamically across individual trajectories. The FELs for MD1–MD3 each showed low-energy conformational basin(s) (Figures 11F–H). However, the distribution and extent of these basins varied among the three landscapes, indicating that the trajectories sampled low-energy conformations but did not produce identical conformational ensembles. Taken together, the three simulations reproduced the overall early-stage adjustment and the broad Rg and RMSF patterns, while differences in RMSD levels, late-stage SASA and hydrogen-bond counts, and FEL profiles reveal replicate-specific variation.

**FIGURE 11:**
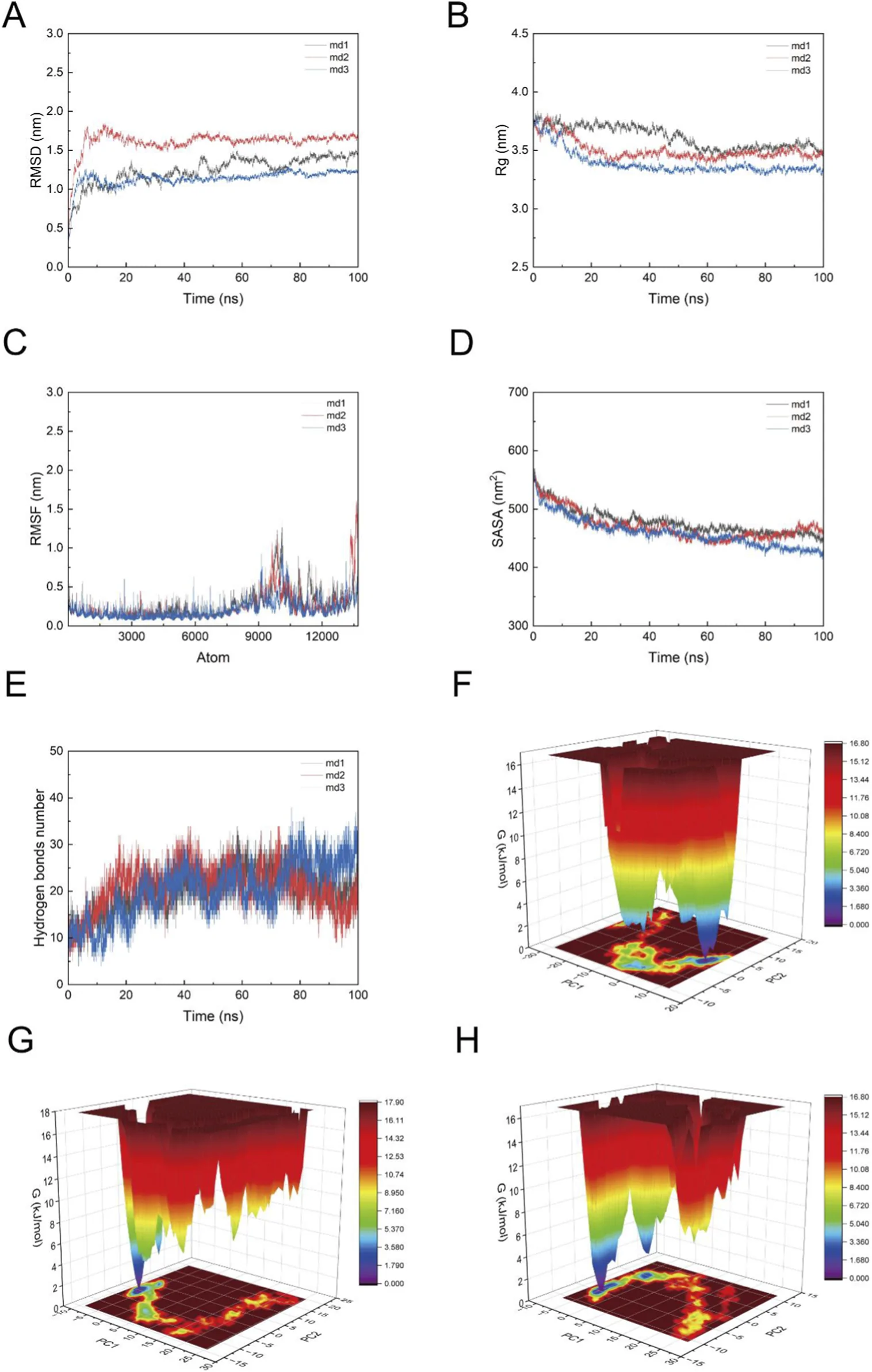
Analysis of Trajectories for Vaccine-Receptor Docking Complexes from Three Independent 100 ns Simulations with Different Initial Velocities. **(A)** RMSD reflects the overall conformational changes of the complex relative to its initial structure during molecular dynamics simulations. When RMSD becomes stable and remains within a relatively small range in the later stages of the simulation, it indicates that the complex has not undergone significant overall structural changes, suggesting good structural stability. **(B)** Rg reflects the overall compactness of the molecule; a stable Rg indicates that the complex does not undergo significant loosening or collapse during the simulation. **(C)** RMSF is applied to assess the flexibility of the vaccine-TLR2 complex. **(D)** SA reflects the solvent-accessible surface area of the vaccine-TLR2 complex. **(E)** The dynamic interaction behavior within the vaccine-TLR2 complex is indicated by the number of hydrogen bonds. **(F,G)** The FEL shows the free energy distribution of the vaccine-TLR2 complex in the conformational space, FEL of the vaccine-TLR2 complex in conformational space from three independent simulations: **(F)** MD1, **(G)** MD2, and **(H)** MD3.

The findings presented in Figure 12 from the iMODS platform include various metrics, including atomic mobility, eigenvalues, variance, covariance, and the elastic network model. Mobility quantifies how flexible the molecule is at each residue. The B-factors obtained experimentally were derived from the relevant PDB field, calculated by multiplying the NMA mobility by (8π^2^). Eigenvalues reflect the amount of energy necessary for structural deformation, with lower eigenvalues signifying greater ease of deformation. Variance is inversely related to eigenvalues, with green denoting cumulative variance and purple individual variance. In the covariance map, red signifies a positive correlation, blue denotes a negative correlation, and white symbolizes the absence of correlation. In the elastic network representation, lighter shades illustrate flexible areas, whereas darker shades indicate more rigid sections.

**FIGURE 12:**
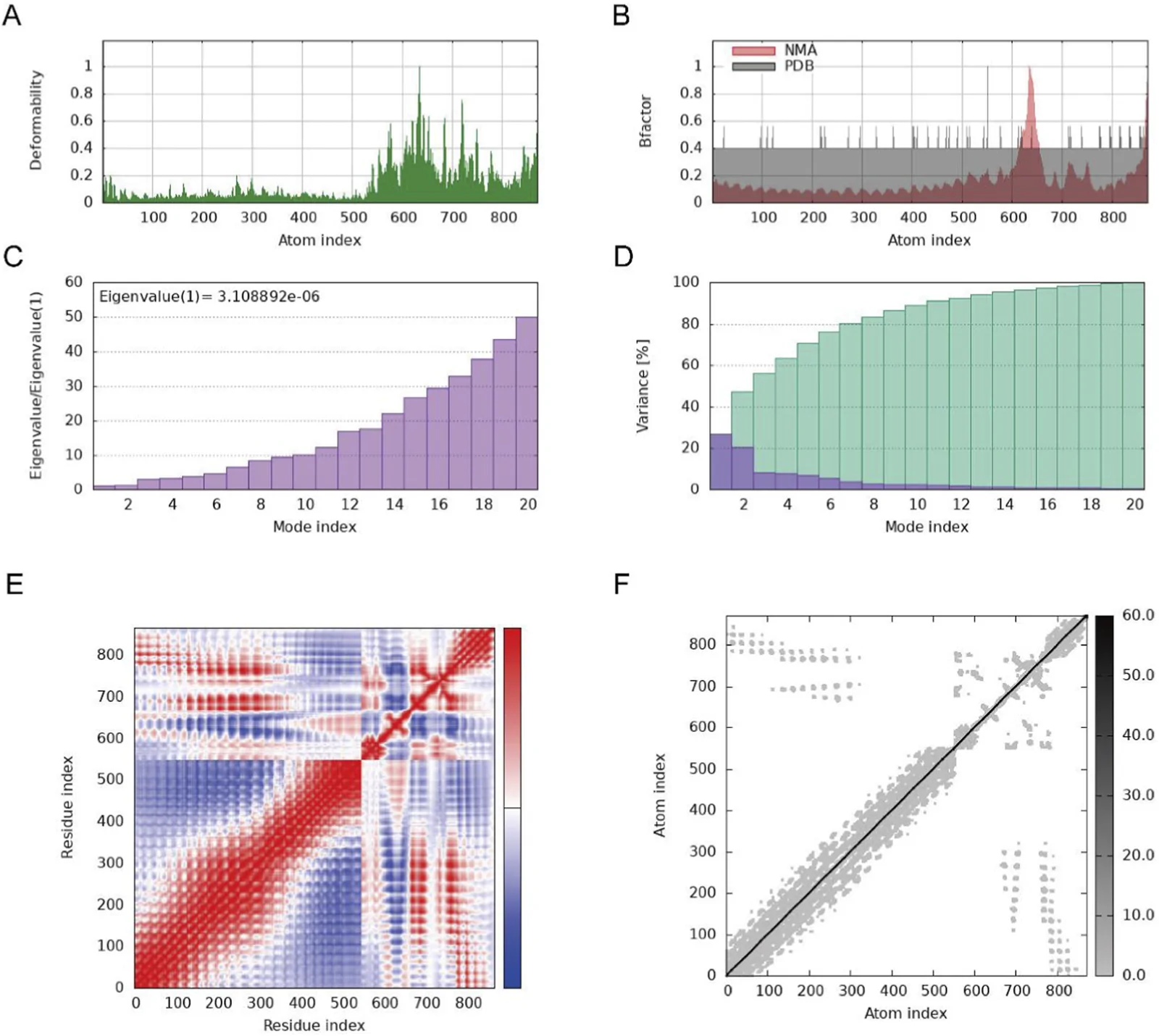
Simulation of molecular dynamics for the vaccine-TLR2 complex was carried out using the iMODS platform. **(A)** Deformability. **(B)** B-factor. **(C)** Eigenvalue. **(D)** Variance. **(E)** Covariance matrix. **(F)** Elastic network.

#### 3.4.9 Prediction of vaccine mRNA

Figure 13 shows the predicted mRNA secondary structure, which was generated by submitting the optimized coding sequence to the RNAfold server. Furthermore, the online server calculated the minimum free energy (MFE) of the two secondary structures. The optimal structure had an MFE of −289.90 kcal/mol, and the centroid structure had an MFE of −306.95 kcal/mol. The predicted stable secondary structure suggests that the optimized coding sequence possesses favorable structural characteristics for efficient transcription and translation in the *E. coli* expression system, potentially facilitating recombinant protein production.

**FIGURE 13:**
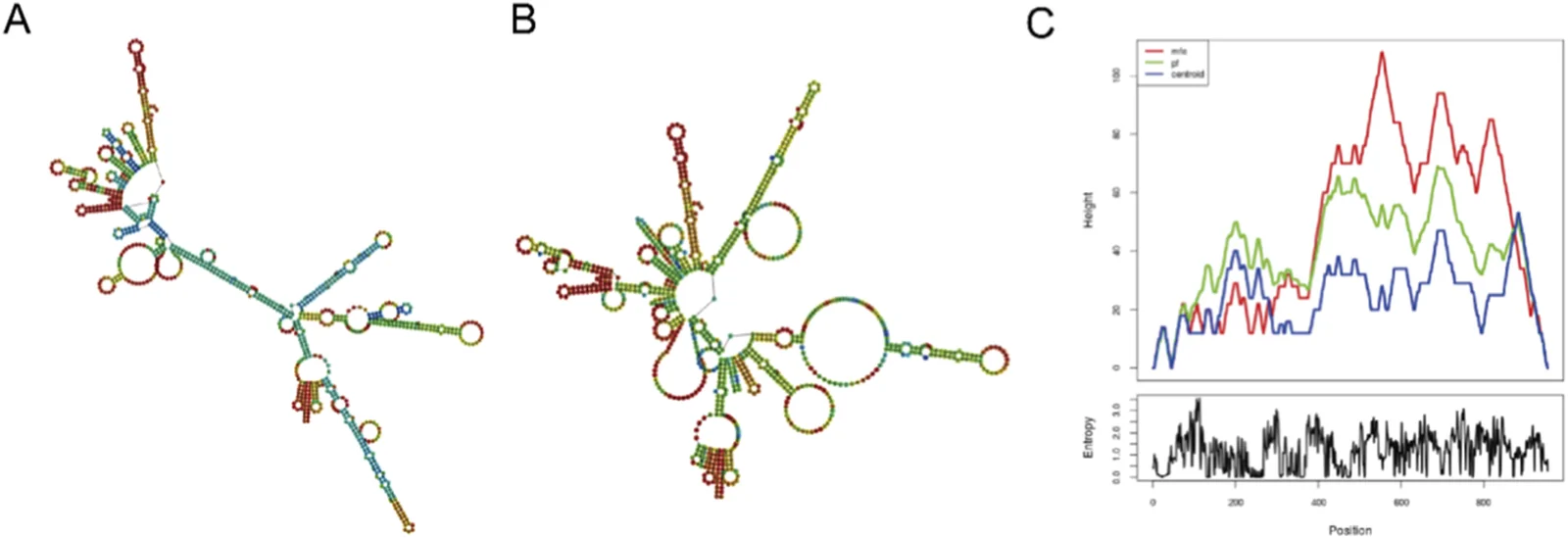
Predicted secondary structural features of the vaccine-encoding mRNA. **(A)** Optimal secondary configuration corresponding to the MFE. **(B)** Centroid-derived structure, with color gradients indicating the probability of individual base-pair formation. **(C)** Mountain plot representation depicting the positional relationships among the MFE structure, the centroid conformation, and the overall thermodynamic ensemble of RNA folding states.

#### 3.4.10 Human proteome homology analysis and prediction

To assess the potential for sequence-based molecular mimicry between the designed vaccine and human proteins, the complete MEV sequence was searched against the *Homo sapiens* Reference protein database. One significant similarity was identified with human beta-defensin 103 precursor (NP_001075020.1), with an E-value of 6 × 10^−24^, 100% sequence identity, and 14% query coverage. Pairwise alignment revealed an exact 45-amino-acid match between residues 1–45 of the MEV construct and residues 23–67 of human beta-defensin 103 precursor, with no gaps. Importantly, this matched region corresponds to the beta-defensin adjuvant incorporated into the MEV construct rather than to the selected vaccine epitopes.

## 4 Discussion

*M. fermentans* is a significant opportunistic pathogen that poses a major health threat to immunocompromised individuals. Typically, *M. fermentans* infections are associated with urogenital tract inflammation and certain types of arthritis, and may also present with nonspecific systemic symptoms. The infection process can be insidious, leading to delayed diagnosis and a prolonged disease course (Benedetti et al., 2014). The immune response triggered by *M. fermentans* infection is often inadequate or transient, failing to establish lasting protective immunity, which predisposes individuals to recurrent or persistent infections. In recent years, the issue of antibiotic resistance in *M. fermentans*—particularly to drugs such as macrolides—has become increasingly prominent (Pereyre et al., 2002). Compounded by its inherent lack of a cell wall, a characteristic that limits the efficacy of traditional antibiotics, this drug resistance poses a serious challenge to clinical treatment and infection control.

Bacteria encounter a wide range of environmental and host-derived stresses during their life cycle and infection, including changes in temperature, pH, osmolarity, nutrient availability, and oxidative conditions (Yan et al., 2024). To cope with these adverse conditions, bacteria activate coordinated stress-response systems involving diverse stress-associated proteins that contribute to cellular homeostasis, protein quality control, and bacterial survival (Njenga et al., 2023; Bouillet et al., 2024). Among these proteins, heat shock proteins (HSPs) constitute a major and highly conserved class of stress-associated molecular chaperones (Wickramaratne et al., 2024). Bacterial HSPs are critical immunogenic components that serve as significant activators of the immune system (Zügel and Kaufmann, 1999b). In the genome of *M. fermentans*, heat shock proteins have been identified as virulence-associated factors; these proteins are typically involved in stress survival, pathogenic processes, and immune evasion (Zella et al., 2018). More importantly, as highly conserved pathogen-associated molecular patterns, they can be effectively recognized by host pattern recognition receptors, such as TLRs, thereby possessing strong potential to activate both innate and adaptive immunity. Consequently, they are regarded as promising candidate targets for vaccine development (Corigliano et al., 2020). However, due to their high degree of conservation, HSPs may cross-react with host self-proteins, potentially triggering autoimmune risks (Pockley, 2003). Our strategy aims to precisely screen for protective epitopes within HSPs that are specific and highly conserved, rather than utilizing the full-length protein, to circumvent potential immunopathological responses induced by non-protective regions. To this end, we performed a BLASTp analysis on the full-length MEV, which revealed only one significant hit: the mature peptide sequence of human beta-defensin 103, which was incorporated as an adjuvant at the N-terminus of the vaccine construct. None of the 15 selected epitopes showed significant sequence identity to human proteins. Nevertheless, the potential immunological consequences of the human-derived adjuvant cannot be completely excluded by *in silico* analysis and warrant further experimental validation. We focus on designing a novel MEV targeting *M. fermentans* stress-associated proteins, with the goal of synergistically eliciting innate as well as adaptive immune responses, thereby enhancing the host’s ability to clear the pathogen while minimizing immune-related side effects caused by non-essential antigenic components.

To conduct the MEV study, we identified six candidate stress-associated proteins: Trigger factor (ADV34158.1), DnaK (ADV34134.1), DnaJ (ADV34142.1), putative heat shock ATP-dependent protease (ADV34408.1), ATPase subunit (ADV35023.1), and thioredoxin reductase (ADV34906.1). A thorough examination and prediction of their antigenicity, solubility, virulence, presence of signal peptides, physicochemical characteristics, secondary structures, and 3D conformations were conducted. Using a variety of online servers, we screened epitopes that exhibited strong antigenicity, high immunogenicity, absence of toxicity and allergenicity, with promising capacity to trigger interferon secretion. The multi-epitope vaccine comprised six B-cell epitopes (linked via KK), four CTL epitopes (joined by AAY), and five HTL epitopes (connected with GPGPG). Furthermore, a beta-defesin was incorporated as an adjuvant at the N-terminus of the vaccine. The beta-defensin 103-derived sequence was incorporated into the MEV construct as an immunomodulatory adjuvant. Previous studies have demonstrated that hBD-3 can promote dendritic cell maturation and enhance T-helper type 1 responses, supporting its potential role as an endogenous adjuvant for immunization. In addition, hBD-3 has been reported to exert context-dependent immunomodulatory effects, including modulation of pro-inflammatory cytokine production (Semple et al., 2010; Ferris et al., 2013; McGlasson et al., 2017).

The analysis of the vaccine’s 2D structure showed that it consists of 33.33% α-helix, 17.30% extended strand, 7.23% beta turn and 42.14% random coil. Research has demonstrated that areas characterized by random coils act as main recognition sites for lymphocytes and antibodies, further indicating that this vaccine possesses strong immunogenic properties (Zhang et al., 2023). Furthermore, the optimized tertiary structure, confirmed by ProSA-web, produced a Z-score of −4.08, supporting its low-energy configuration (Andongma et al., 2023). For high-yield expression and purification, the vaccine sequence was successfully inserted into the pET-28a (+) vector. An immunoinformatics simulation was carried out using the C-ImmSim server, and the computational assessment of the vaccine’s immunogenic potential showed a strong immunostimulatory response, as indicated by heightened antibody levels, increased cytokine production, and a rise in the count of antigen-presenting cells. After the final design, the vaccine underwent docking with TLR2, and an MD simulation was conducted on the vaccine-TLR2 complex. The findings suggested that this complex demonstrates favorable stability and a high level of compactness.

Several limitations of the present study warrant acknowledgment and should be addressed in future investigations. First, all computational prediction algorithms inherently possess varying degrees of inaccuracy. This issue may be partially mitigated by adopting a consensus-based approach that integrates the outputs of multiple algorithms performing the same predictive task, thereby enhancing the overall reliability of the predictions. Second, the actual protective efficacy of the candidate vaccine constructs has not yet been experimentally validated. To address this, subsequent studies should incorporate animal model experiments to systematically evaluate and confirm their protective capacity *in vivo.* Third, the current RV pipeline is primarily designed to identify proteinaceous antigens and consequently fails to recognize carbohydrate-based and glycolipid-based antigenic determinants. This inherent bias may lead to the inadvertent omission of potentially immunogenic non-protein antigens, underscoring the need for complementary screening strategies to achieve a more comprehensive antigen discovery process. Fourth, The AlphaFold confidence results warrant caution when interpreting the docking and MD analyses. The relatively low and regionally variable pLDDT scores, together with elevated PAE values for many nonadjacent residue pairs, may be related to the artificial architecture of the MEV, which combines epitopes and functional components that do not form a continuous naturally evolved protein domain. Flexible linker regions may further contribute to uncertainty in the relative arrangement of these components. This is a possible explanation for the confidence pattern rather than a cause established by the present analysis. Favorable Ramachandran, ProSA, and ERRAT results after refinement support the geometric or model-quality assessment of the refined structure, but do not establish the accuracy of its global fold. Therefore, the docking and MD findings should be interpreted as model-dependent computational results.

## 5 Conclusion

M. fermentans, an important opportunistic pathogen, readily infects immunocompromised individuals. A key issue is the lack of a specific vaccine against it. In response, this study constructed an stress-associated proteins-based MEV characterized by high antigenicity, non-toxicity, and non-allergenicity. Compared with conventional vaccine development strategies, reverse vaccinology may help identify candidates with the potential to elicit strong immune responses. Bioinformatic analyses and molecular dynamics simulations suggest that the candidate has favorable predicted structural stability and potential immunogenicity; these computational findings require experimental validation. In summary, this vaccine has the potential to be a candidate vaccine for *M. fermentans*. However, its safety and protective efficacy still require validation through *in vitro* experiments.

## Data Availability

The original contributions presented in the study are included in the article/Supplementary Material, further inquiries can be directed to the corresponding author.
