# Supplementary material for: Rational design of a multi-epitope vaccine targeting the stress-associated proteins for Mycoplasma fermentans via immunoinformatics
Source: Front Bioinform. 2026 Oct 21;6:1994081. doi: 10.3389/fbinf.2026.1994081 (PMC13731115; doi:10.3389/fbinf.2026.1994081)
Supplement: Supplementary file 1 [file Table1.docx]

Epitope conservation: We performed additional BLASTp analyses to evaluate the conservation of each selected epitope across available Mycoplasma fermentans strains. Each selected epitope sequence was individually queried against the available M. fermentans sequences using BLASTp. A total of seven Mycoplasmopsis fermentans strains are currently deposited in the NCBI database. The 15 selected epitopes were individually queried against the NCBI non-redundant protein database using BLAST, with the organism parameter restricted to Mycoplasmopsis fermentans (taxid:2115). The final search results showed that proteins with 100% query coverage and 100% sequence identity matched six out of the aforementioned seven strains, yielding a coverage rate of 85.7%. The analysis showed that the selected epitopes exhibited high sequence conservation among the available strains, supporting their potential to represent conserved antigenic regions of M. fermentans.

| Epitope | Strain | Per.ident | Query Cover | Conclusion |
| --- | --- | --- | --- | --- |
| LVEKILNRKPNKSVNP | JER, M64, MF-I1, MF-I2, NCTC10117, PG18 | 100% | 100% | conservative |
| GDKIKIDGYGEKGRNG | MF-I1, MF-I2, M64, PG18, NCTC10117, JER | 100% | 100% | conservative |
| PFDGGEAQGFDLKLGS | PG18, JER, M64, NCTC10117, MF-I1, MF-I2 | 100% | 100% | conservative |
| KEVAKEYNEENEKTKK | PG18, MF-I2, JER, MF-I1, NCTC10117, M64 | 100% | 100% | conservative |
| HMLIGTGRNAEGGMDA | PG18, MF-I2, JER, MF-I1, NCTC10117, M64 | 100% | 100% | conservative |
| GENQLTSVTIRNKKTN | PG18, MF-I2, JER, MF-I1, NCTC10117, M64 | 100% | 100% | conservative |
| KIPGFRPGK | PG18, MF-I2, JER, MF-I1, NCTC10117, M64 | 100% | 100% | conservative |
| KFAFRILSL | PG18, MF-I2, JER, MF-I1, NCTC10117, M64 | 100% | 100% | conservative |
| EINGKEWEK | PG18, MF-I2, JER, MF-I1, NCTC10117, M64 | 100% | 100% | conservative |
| LVGEPGVGK | PG18, MF-I2, JER, MF-I1, NCTC10117, M64 | 100% | 100% | conservative |
| LGTTNSAVAIVDGGT | PG18, MF-I2, JER, MF-I1, NCTC10117, M64 | 100% | 100% | conservative |
| ELVYTLEINGKEWEK | PG18, MF-I2, JER, MF-I1, NCTC10117, M64 | 100% | 100% | conservative |
| QAGLKKEQFNIDDKT | PG18, MF-I2, JER, MF-I1, NCTC10117, M64 | 100% | 100% | conservative |
| IEVSSFKSKNSGIKL | PG18, MF-I2, JER, MF-I1, NCTC10117, M64 | 100% | 100% | conservative |
| IKDRFENYHNVKIQD | PG18, MF-I2, JER, MF-I1, NCTC10117, M64 | 100% | 100% | conservative |

Confidence in the AlphaFold structural model: We have therefore re-evaluated the AlphaFold structural model using the per-residue predicted Local Distance Difference Test (pLDDT) scores and predicted aligned error (PAE) metrics. The overall pLDDT confidence of the MEV model was relatively low and varied among different regions of the construct. This relatively low confidence is considered in the context of the artificial nature of the MEV. Unlike naturally occurring proteins, the MEV was constructed by combining multiple selected epitopes, linkers, and additional functional components based on reverse vaccinology. Such an artificially assembled sequence does not necessarily possess the evolutionary constraints and naturally optimized folding patterns of native proteins. Therefore, uncertainty in the global arrangement of individual components and the flexibility of linker regions can result in relatively low structural prediction confidence.
